# Risk Behaviors among Migrant Adolescents in Italy

**DOI:** 10.3390/children10111816

**Published:** 2023-11-15

**Authors:** Emanuele Koumantakis, Rosanna Irene Comoretto, Paola Dalmasso, Michela Bersia, Patrizia Lemma, Giacomo Lazzeri, Paola Nardone, Alessio Vieno, Tommaso Galeotti, Paola Berchialla, Lorena Charrier

**Affiliations:** 1 Department of Public Health and Pediatrics, University of Torino, 10126 Torino, Italy; emanuele.koumantakis@unito.it (E.K.); rosannairene.comoretto@unito.it (R.I.C.); michela.bersia@unito.it (M.B.); patrizia.lemma@unito.it (P.L.); lorena.charrier@unito.it (L.C.); 2 Post Graduate School of Medical Statistics, University of Torino, 10126 Torino, Italy; 3 Department of Molecular and Developmental Medicine, University of Siena, 53100 Siena, Italy; giacomo.lazzeri@unisi.it; 4 National Centre for Disease Prevention and Health Promotion, Istituto Superiore di Sanità (National Institute of Health), 00161 Rome, Italy; paola.nardone@iss.it; 5 Department of Developmental Psychology and Socialisation, University of Padova, 35131 Padova, Italy; alessio.vieno@unipd.it (A.V.); tommaso.galeotti@phd.unipd.it (T.G.); 6 Department of Clinical and Biological Sciences, University of Torino, 10043 Orbassano, Italy; paola.berchialla@unito.it

**Keywords:** adolescents, migrants, risk behaviors, substance use, gambling

## Abstract

Adolescence is a critical period for engaging in health risk behaviors. Migrant adolescents may face unique challenges due to acculturation stress. This study aims to monitor substance use and problem gambling among migrant adolescents living in Italy. Data from the 2017/18 Health Behavior in School-Aged Children survey in Italy were analyzed. The 18,794 participants included 15-year-olds, categorized as native or migrants, with ethnic backgrounds from Western, Eastern European, or non-Western/non-European countries. Girls had higher smoking rates, while boys exhibited higher prevalence of alcohol-related risk behaviors, cannabis use, and gambling. Boys from Eastern European countries displayed a greater risk of drunkenness (OR: 1.58, 95% CI: 1.06–2.37), particularly in the first generation, while those from Western countries showed a higher risk of multiple substance use (OR: 1.44, 95% CI: 1.05–1.96). Girls from Eastern European and non-Western/non-European countries had a lower risk of alcohol consumption (OR: 0.50, 95% CI: 0.29–0.85; OR: 0.55, 95% CI: 0.33–0.91, respectively). Finally, boys, especially those from Eastern European and non-Western/non-European countries, had a significantly higher risk of problem gambling (OR: 1.83, 95% CI: 1.04–3.22; OR: 2.10, 95% CI: 1.29–3.42, respectively). This disparity was more pronounced in the first generation, possibly due to acculturation challenges and socio-economic factors. Risk behaviors in adolescents are influenced by complex interplays of gender, cultural factors, and migration generation. Preventive strategies should consider these factors to effectively address substance use and gambling in this heterogeneous population.

## 1. Introduction

Adolescence is a crucial phase in an individual’s physical, cognitive, emotional, and social development. During this period, young people usually have their first experiences with substance use, as approximately three out of five 15-year-olds have already consumed alcohol, one out of four have smoked, and one out of seven used cannabis [1,2,3]. Moreover, it has been observed that adolescent substance use can lead to serious and persistent negative consequences [4,5,6]. Adolescence is also a high-risk period for gambling disorders, with an estimated prevalence of around 2.6% in Italy [7].

Previous studies suggest that health risk behaviors tend to cluster together, a notion that stems from evidence that adolescents usually exhibit a co-presence of several risk behaviors [8,9,10,11,12,13,14,15]. The association between gambling and substance use has been well described [14,16]. In particular, Italian studies on adolescents and young adults have indicated that over half of the individuals with gambling problems had smoked, and four out of five had consumed alcohol at least once in the previous month. Moreover, moderate-risk/problem gamblers exhibited an approximately two-fold higher risk of tobacco smoking and alcohol consumption compared to those without gambling problems [17,18]. Even when considering different substances, it has been observed that an adolescent habitual smoker is more likely to engage in alcohol and cannabis use, and binge drinking [15].

Moreover, it is recognized that the social context in which people grow up and interact plays a crucial role in the development of risk behaviors [19]. In fact, while family and social support are known to protect adolescents against substance use and gambling [20,21,22], young people are still significantly influenced by the behaviors and attitudes of their friends [23].

A subgroup of adolescents that may be particularly susceptible to engaging in such risk behaviors is that of immigrants since the migration process could be associated with higher rates of health risk behaviors due to the high level of stress during both the actual displacement phase and the so-called “acculturation” process [24]. In fact, acculturation has been associated with a higher prevalence of substance use than native peers [25,26,27,28,29]. The same phenomenon has been observed for gambling [30], as acculturation stress increases the likelihood of frequency and severity of problem gambling [31].

However, despite lower economic and social resources and the stress associated with acculturation, several studies found that adolescents from immigrant groups demonstrate better academic, behavioral, and health outcomes than natives [32,33,34]. Important factors that appear to play a role in this context are greater ethnic pride and adherence to traditional family values, which mitigate the influence of negative characteristics of the dominant host culture [33,35].

The above theories seem to apply mainly to first-generation immigrants, that is, those born abroad. Indeed, the protective role determined by the persistence of the customs of the area of origin tends to disappear among second-generation immigrants, who were born in the host country and have at least one foreign-born parent, and who are therefore more at risk of conforming to the norms of the host country [34,36]. First-generation adolescents appear to be at lower risk for externalizing problems, such as substance use, than their second-generation peers; they tend to hang out less with native peers and, consequently, they experience less peer pressure for these risk behaviors [37]. On the contrary, the evidence from the literature shows higher rates of at-risk/problem gambling among first-generation immigrants compared to native peers or adolescents from other immigrant generations [22,38,39].

Currently, the literature on risk behaviors among migrant adolescents is still scarce, and migrants are often considered as a single population. However, a comparison with the native population cannot overlook the extreme diversity of cultures in their respective countries of origin. In Italy, students without Italian citizenship are increasing, being almost 10% in 2018, and therefore provide good material with which to make such comparisons [40].

In this context, the present work aims to monitor substance use and problem gambling among migrant adolescents living in Italy, considering the different areas of origin to provide appropriate guidance for the design of targeted policies and interventions. In addition, as it is widely described in the literature that the prevalences of substance use and gambling are different among boys and girls [41,42,43,44], separate analyses for the two genders should allow for a broader and more detailed understanding of the phenomenon. Finally, we seek to provide a perspective on migrants’ risk behaviors that takes into account the migrant generation, which is an increasingly common distinction in migration research [6,22].

## 2. Materials and Methods

The Italian data from the 2017/18 Health Behavior in School-Aged Children (HBSC) study have been used. HBSC is a World Health Organization Collaborative Cross-National Survey, conducted every four years to gather information on the health and behaviors of 11-, 13- and 15-year-olds. In 2018, a national survey was carried out in Italy on a representative sample of adolescents from all regions. The participants completed standardized, self-administered, and anonymous questionnaires about, among others, their smoking habits, alcohol consumption, drunkenness, and binge drinking, as well as cannabis intake and gambling. The country of birth of participants and their parents was used to determine the ethnic background of each adolescent. As the questions related to cannabis intake and gambling were asked only to 15-year-old students, all analyses were restricted to this age group (18,794 subjects). Participation was voluntary, and parental consent was obtained through an opt-out process. A recognized ethics committee approved the national research protocol. For more information on the HBSC study and its Italian component, refer to the paper by Lazzeri et al. [45].

The risk behaviors investigated in the present study included:

- *Current smoking*. Smoking habit was assessed by asking participants how many days they had smoked cigarettes in the past 30 days. Responses ranged from 0 to every day. Under HBSC protocol, if the answer was at least one day, participants were considered current smokers [1].
- *Current weekly drinking*. Students were asked how many days they had drunk in the last 30 days. Responses ranged from “Never” to “Every day” and were dichotomized into “at least weekly” (i.e., weekly or daily) vs. “monthly, rarely or never” [1].
- *Drunkenness.* Adolescents responded to the question, “Have you ever had so much alcohol that you were really drunk?” The response options were “No, never” (1), “Yes, once” (2), “Yes, 2–3 times’’ (3), “Yes, 4–10 times” (4), and “Yes, more than 10 times” (5). Students were considered having this risk behavior if they reported getting drunk at least twice in their lifetime [1].
- *Binge drinking.* This risky behavior has been investigated in the Italian national HBSC survey since 2010. The binge drinking category asks students to indicate whether they had drunk five or more glasses of alcohol on a single occasion in the past 12 months. Responses were dichotomized into “Yes, at least once” (i.e., binge drunk once or more times) and “No, never” [46].
- *Cannabis use.* Participants were asked if they had ever taken cannabis in their lifetime, with response options ranging from “Never” to “30 days or more”. Adolescents were considered cannabis users when they answered that they had taken cannabis for at least 3 days in their lifetime [1].
- *Multiple substance use.* Students reporting two or more among current cigarette smoking, alcohol consumption, and cannabis consumption were considered multiple substance users [47].
- *At-risk or problem gambling.* The 12-item South Oaks Gambling Screen-Revised for Adolescents (SOGS-RA), which is the most used screening tool for assessing problem gambling in adolescents, was administered. The scoring was: 0–1, “No problem gambling”; 2–3, “At-risk gambling”; and 4 or more, “Problem gambling”. Those categories were then dichotomized into “At risk-problematic gambling” and “No problem gambling”, as in literature, at-risk and problem gamblers seem to exhibit similar characteristics [22,48,49,50,51].

The explanatory variables considered were:

- *Gender.* Participants were asked to indicate whether they were a boy or a girl.
- *Generation of migration.* Adolescents were classified as “natives” if both parents were born in Italy, otherwise, they were considered migrants. Specifically, they were classified as “first-generation immigrants” if they were born abroad and at least one parent was born abroad, or as “second-generation immigrants” if they were born in Italy and at least one parent was born abroad [52].
- *Area of origin.* Based on the country of birth of the mother (or, if missing or born in Italy, on the country of birth of the father), the ethnic background was categorized into three different areas: “Western Countries”, “Eastern European Countries”, and “non-Western/non-European Countries” [52,53].

The following is a list of the nations included in each area:

  1. “Western Countries”: European Union (EU)—14 countries (member states prior to May 2004), and United Kingdom, Switzerland, Norway and Iceland. It also includes United States, Canada, Australia, and New Zealand, all classified by the International Monetary Fund as advanced economies countries [54];
  2. “Eastern European countries”: EU—13 countries (new member states joining the EU after May 2004), plus Albania, Bosnia, Macedonia, Moldavia, Serbia, and Ukraine [54];
  3. “Non-Western/non-European countries”: countries in Africa, South or Central America, and Asia. As immigrant youth from non-Western/non-European countries showed numerous similarities with the other migrants’ area groups, they were combined into a single group.

The relatively low number of immigrants did not allow stratification by country of origin.

- *Peer substance use-related variables*. The HBSC questionnaire asked for the number of friends who smoke, drink alcohol, or take cannabis. For each of the three substances, a variable was created categorizing the number of friends using those substances into three levels: none/a few, some, many/all [1].
- *Support-related variables*. Referring to the 2017/18 HBSC Research Protocol [1], family and social support items were used to create the following variables: high *family support*, high *friends support*, high *teachers support*, and high *classmates support*”.

Descriptive data are shown as absolute frequencies and percentages for categorical variables. Multivariable logistic regressions were run to model the odds ratios of risk behaviors of migrant adolescents with different ethnic backgrounds compared to their native counterparts [55]. Models were stratified by gender and adjusted for other substance use, peer substance use, and family, friends’, teachers’, and classmates’ support when the dependent variable was smoking, alcohol, drunkenness, binge drinking, or cannabis use. Analyses for multiple substance use and gambling as the dependent variable were not adjusted for peer multiple substance use and problematic gambling since these items were not included in the Italian HBSC survey. All statistical tests were two-sided, and the level of statistical significance was set at 0.05. All analyses were carried out using Stata/SE 17.0 [56].

## 3. Results

The study included 18,794 15-year-old students, with 9345 boys (49.7%) (Table 1). In both genders, the majority of the students was Italian (around 84%), while the remaining individuals were equally distributed among Western, Eastern European and non-Western/non-European countries. More in detail, considering the non-Western/non-European area, 365 (35.1%) adolescents were migrants from Central and South America, 327 (31.47%) from North Africa and the Middle East, 235 (22.6%) from Asia and the Far East, and 112 (10.8%) from sub-Saharan Africa. A higher prevalence of current smoking was observed among girls than boys (32.4% vs. 26.6%). On the other hand, boys showed current alcohol consumption, drunkenness, binge drinking, cannabis, and at-risk/problem gambling more frequently than girls. Multiple substance use was equally frequent among the two genders (27–28%) and showed similar frequencies in different areas of migration. Migrant students from non-Western/non-European countries had the lowest prevalences for alcohol-related variables, which was also seen in girls for multiple substance use (23.7%). However, boys from this area consumed cannabis (20.6%) and were at-risk/problem gamblers (25.8%) the most. A high percentage (54%) of missing values was found for the gambling item, significantly higher among girls (69%, *p* < 0.001) and in the first generation of migrants (71% vs. 53% of natives and 55% of migrants of second generation, *p* < 0.001). Migrants from non-Western/non-European countries (63%) and Eastern European countries (61%) showed the highest proportion of missing values (*p* < 0.001).

**Figure 1:**
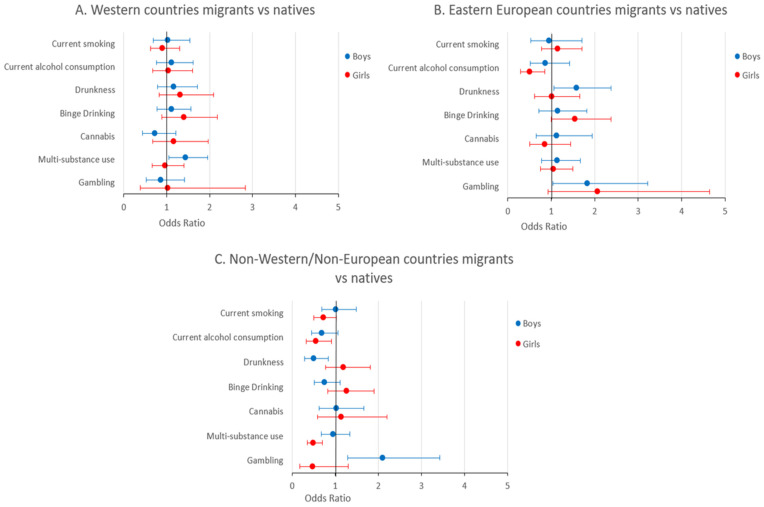
Forest plot illustrating the odds ratios (represented as points) and corresponding 95% confidence intervals (represented as bars) of risk behaviors among migrant students from Western countries (**A**), Eastern European countries (**B**), and non-Western/non-European countries (**C**), compared to their native peers. Different regression models were performed for boys (blue) and girls (red).

The adjusted risk of the investigated behaviors among migrants from different areas of origin compared to their native peers are shown in Table 2 and Figure 1; Table 3 shows the adjusted odds ratios for migrants of first and second generation.

Current smoking, binge drinking, and cannabis use showed no statistically significant differences between migrants and natives; the same was found for current alcohol consumption among males, and drunkenness and gambling among females. Boys from Western countries turned out to be at significantly higher risk than their Italian peers for multiple substance use (OR: 1.44, 95% CI: 1.05–1.96), and those from Eastern European countries for drunkenness (OR: 1.58, 95% CI: 1.06–2.37) and problem gambling (OR: 1.83, 95% CI: 1.04–3.22). Students from non-Western/non-European countries were also at a significantly higher risk of problem gambling (OR: 2.10, 95% CI: 1.29–3.42) but had a significantly lower risk of drunkenness compared to Italians (OR: 0.50, 95% CI: 0.29–0.84). Girls from Eastern European countries and non-Western/non-European countries were at lower risk of current alcohol use than Italian peers (OR: 0.50, 95% CI: 0.29–0.85; OR: 0.55, 95% CI: 0.33–0.91, respectively), as were females from non-Western/non-European countries for multiple substance use (OR: 0.49, 95% CI: 0.35–0.70).

Having a few friends who use one of the substances significantly increased the risk of consuming the same substance, and the increase was even greater if many friends used it. In contrast, family and teacher support reduced the risk of substance use and problem gambling in almost all analyses. Friend support was associated with an overall increase in substance use and problem gambling, while classmate support showed an uncertain role.

## 4. Discussion

This study provides an overview of substance use and gambling among adolescents in Italy, focusing on differences between natives and migrants according to their origin and cultural background.

Our results highlight a higher prevalence of current smoking among girls compared to boys, confirming the Italian trend of a reversal in frequency between the two genders [15]. This trend, in turn, distinguishes itself from the overall European trend, which instead shows an increasingly greater smoothing of the aforementioned differences [2]. Nevertheless, substantial differences between Italians and migrants from different areas of origin did not emerge. This suggests that regarding cigarette smoking, habits from the country of origin tend to level out and resemble those of the host country. This phenomenon has also been observed in adults and has been found to be associated with the increasing time since migration [57].

In our opinion, alcohol-related risk behaviors require more complex interpretation. In general, alcohol consumption, drunkenness, and binge drinking were less frequent among girls, in line with European data [2], and among migrants from non-Western/non-European countries. However, when modeling risks of engaging in the aforementioned risk behaviors, boys from this area seem to be protected in comparison with natives, and girls showed a non-significant increased risk of drunkenness and binge drinking. However, the risk of binge drinking became more than double, and statistically significant, when considering first-generation migrants. The coexistence of numerous cultures in this area of origin makes the interpretation of these results complex. However, one can imagine that acculturation stress plays a role in increasing the risk of first-generation migrant adolescents, considering that their culture is often very different from that of the host country. On the other hand, greater family cohesion and adherence to home country traditions may play a protective role against substance use. This may be true for areas of North Africa, the Middle East, and parts of sub-Saharan Africa, where the predominant religion is Islam, which prohibits alcohol consumption [6,58]. In contrast, the non-Western/non-European group also includes migrants from Central and South America, regions where substance use is still a concern, despite a decline in recent years [59]. Indeed, when comparing migrants from North Africa/Middle East, Central/South America, and Asia/Far East, significant differences emerged in terms of prevalences of the investigated risk behaviors. Specifically, alcohol consumption, binge drinking, and drunkenness turned out to be more frequent among students from Central/South America. However, in general, what emerged is a protective effect that can be attributed to the culture and religion of the country of origin, despite the fact that this effect seems to be lower in the first generation of migrants, likely due to acculturative stress. On the other hand, boys from Eastern European countries showed a higher risk of drunkenness, and likewise for girls with binge drinking compared to natives. This trend is most prominent in the first generation of migrants. Historically these countries have high rates of alcohol consumption and heavy drinking [60,61], so our results may align with the theory that first-generation immigrants adopt behaviors similar to those in the country of origin, while second-generation immigrants tend to adapt to those in the host country. However, a recent study on 2014 HBSC data [62] found no significant differences in alcohol use between native Italian and native Romanian adolescents, who are the most represented group from Eastern Europe in our country. Even in less represented Eastern European countries, drinking habits among young people have been decreasing in the last decade [63]. Therefore, rather than adherence to traditions from the country of origin, it seems that the increased risk of drunkenness and binge drinking may be determined by acculturative stress, which primarily affects first-generation migrants and decreases in the second one [25,26,62].

When considering cannabis use, less than one out of six adolescents consumed it for at least three days in their lifetime. Boys showed a higher prevalence, while differences between natives and migrants were not evident, with the only exception of boys from non-Western/non-European countries, who showed the highest prevalence. Again, migrants appear to share similar risks of engaging in this risk behavior with native peers, although European studies show conflicting evidence [6,64]; while some studies are in line with our results, others show increased or even decreased prevalence of cannabis use among migrants [6]. In particular, two studies conducted in Spain [65] and Norway [66], that considered migrants from Muslim regions separately, showed a lower prevalence of cannabis use compared to non-migrant peers. As a consequence of these conflicting findings, no acculturative hypothesis or cultural protective role influence on this risk behavior during adolescence has been proven yet [6].

Similarly to cannabis, the prevalence of multiple substance use was similar among immigrants and natives, as well as in the two genders. Only girls from non-Western/non-European countries consumed at least two substances in the last month less frequently, consistent with the lower prevalence of current alcohol use described above. This results in a decreased risk of multiple substance use compared to native girls, which seems to be more pronounced in the second generation of migrants. In contrast, boys from Western countries showed a higher risk of engaging in this composed risk behavior, which was characteristic of the second generation. This result seems to contradict the acculturation theory, which suggests that the first generation of immigrants might be more exposed to unhealthy behaviors than the second generation, which is generally considered more acculturated [25]. In this sense, our findings provide new insights into multiple substance use in migrant adolescents, suggesting that the underlying mechanisms might be different from those related to smoking, alcohol, or cannabis use. Since most studies consider them separately, it is desirable that further studies evaluate this behavior to define risk or protective factors and appropriate interventions against it.

Approximately one in eight adolescents exhibited at-risk/problem gambling behavior, with boys having a prevalence more than twice than girls. Eastern European immigrants were found to gamble more than natives, as well as boys from non-Western/non-European countries, who also had the highest prevalence, with around one out of four adolescents at-risk/problem gamblers. Some specific risk factors related to gambling need to be considered in order to understand these differences. In particular, since immigrants frequently find themselves in a worse socio-economic condition, their desire for redemption can lead to an increased predisposition to engage in such behavior [22,67,68]. On the other hand, gambling can provide an opportunity for immigrants who have lost their social connections to socialize and rebuild their networks [69,70]. When combined with the effect of acculturation stress, the aspects described above may explain the differences and the corresponding increase in gambling risk observed among boys from Eastern European and non-Western/non-European areas compared to natives, which mainly affects the first generation in both cases and decreases slightly in the second. This is in line with previous research on 2013/2014 HBSC data [22] and other non-European studies [38,39], which found a higher risk of gambling in the first generation than in the second-generation migrant adolescents. However, our results should be read with caution because of the large number of missing values, which are probably not random. In fact, out of a slightly lower proportion of missing (55%), females seem less likely to respond, as do immigrants from Eastern European countries or non-Western/non-European countries. Finally, the higher proportion of non-responders in students who were born abroad could be partly explained by the fact that the questionnaires were proposed only in Italian.

Prevalences of high teacher and family support were higher among boys than girls. While no differences were evident between migrants and natives for teacher support, lower prevalences of family support was found among adolescents coming from non-Western/non-European countries and girls from Eastern European countries. Teacher and family support are confirmed to play an important role in preventing unhealthy habits [20,21,22,66,71,72], as in our analyses were strong protective factors for each risk behavior, independent from the area of origin considered. This suggests that substance use prevention should be carried out through not only school-based but also family-oriented interventions [21,73,74] and can potentially be effective in adolescents with an immigration background. Some authors argue that family connectedness contributes to the protective role of adherence to the country of origin’s habits against risk behaviors, as second-generation immigrants show a deterioration of those old cultural and family values and attitudes in favor of new ones [75], thus increasing susceptibility to substance use [32,37]. In this line, enhancing family presence and commitment could mitigate these negative behaviors deriving from acculturation [76].

This study draws on data from the Italian HBSC survey, which boasts validated questions and a very large number of students attending schools throughout Italy. Consequently, our materials are of high quality and extensive, allowing for an evaluation of the phenomenon of risk behaviors among native and migrant populations at a national level. However, there are some limitations. Firstly, comparisons between migrants and non-migrants may be affected by a large difference in sample size between the two groups, as native-born Italians account for nearly 85% of the total sample. Second, the power of the analyses is progressively reduced by stratifying first by gender and then by generation, resulting in less precise estimates. Third, within the migrant group from the non-Western/non-European area, many cultures, including very different ones, are considered, making it less homogeneous and difficult to interpret. Fourth, it is important to recognize that the influence of peer groups also plays a significant role in adolescents’ substance use [23]. Unfortunately, it was not possible to account for this factor as a confounding variable in our analyses of multiple substance use because it was not addressed in the Italian HBSC questionnaire [45]. Fifth, the large number of nonrandom missing values in items related to gambling may increase the bias when assessing the association between migrants’ origin and this risk behavior. Sixth, the characteristics of non-respondents were not collected, which could affect the representativeness of the sample of Italian 15-year-old adolescents, especially immigrants due to cultural differences. However, since the response rate was 97.1%, we can assume that our sample was sufficiently representative of Italian 15-year-olds, including the migrant population [77]. Finally, a factor that may be relevant in the migrant acculturation process, especially for first-generation immigrants, is the length of stay in the host country [78] or age at migration [79]. Unfortunately, in the context of the HBSC survey, this question is not included, thus this potential confounding variable remains unmeasured.

## 5. Conclusions

In conclusion, our study highlights the heterogeneity of risk behaviors among migrants compared with the habits of adolescents in the host country. Acculturation stress and adherence to home country traditions seem to be the main factors involved in the balance of substance use and at-risk or pathological gambling. In addition to gender and migration generation, policies aimed at preventing the occurrence of risk behaviors in adolescents should consider the cultural and traditional diversity of migrants based on their origin. Further studies on risk behaviors among migrant adolescents should consider the area of origin as a potential confounding factor.

## Figures and Tables

**Table 1:** Characteristics of the Italian 2018 HBSC study’s student sample. Relative (%) frequencies are reported for qualitative variables, after stratification by gender and area of origin. Abbreviations: W, Western countries; EE, Eastern European countries; nW/nE, non-Western/non-European countries.

	BOYS					GIRLS				
	AREA OF ORIGIN					AREA OF ORIGIN				
	Italy	W	EE	nW/nE	Total	Italy	W	EE	nW/nE	Total
	N = 7862	N = 477	N = 474	N = 532	N = 9345	N = 7953	N = 496	N = 493	N = 507	N = 9449
Current smoking	26.8%	26.2%	24.3%	25.6%	26.6%	32.7%	34.1%	31.8%	27.2%	32.4%
Current alcohol consumption	29.4%	31.1%	24.1%	17.6%	28.5%	20.2%	22.1%	15.2%	12.8%	19.6%
Drunkenness	20.4%	21.0%	23.4%	16.3%	20.3%	18.4%	21.7%	20.2%	15.6%	18.5%
Binge drinking	27.2%	27.4%	24.0%	19.6%	26.6%	21.5%	24.7%	22.7%	20.2%	21.7%
Cannabis	16.9%	17.7%	17.6%	20.6%	17.1%	12.0%	15.4%	13.7%	13.6%	12.3%
Multiple substance use	27.4%	27.5%	30.6%	27.3%	27.5%	28.4%	31.5%	28.6%	23.7%	28.3%
At risk/problem gambling	17.4%	16.6%	21.6%	25.8%	17.9%	6.8%	7.6%	15.2%	7.6%	7.2%
Has friends who smoke										
None/Few	37.2%	30.6%	37.9%	42.0%	37.1%	31.6%	28.3%	29.2%	32.9%	31.4%
Some	34.0%	37.3%	30.5%	28.1%	33.8%	31.2%	33.7%	27.1%	32.2%	31.2%
Many/All	28.8%	32.2%	31.6%	30.0%	29.2%	37.2%	38.0%	43.6%	34.8%	37.4%
Has friends who drink alcohol										
None/Few	27.6%	24.4%	30.7%	33.3%	27.8%	25.6%	23.4%	23.7%	27.1%	25.5%
Some	33.1%	33.7%	31.8%	27.0%	32.8%	32.4%	33.1%	30.2%	30.9%	32.3%
Many/All	39.3%	41.9%	37.6%	39.6%	39.4%	42.0%	43.5%	46.0%	42.0%	42.3%
Has friends who take cannabis										
None/Few	71.0%	69.9%	71.1%	64.8%	70.7%	70.7%	67.8%	67.3%	68.6%	70.3%
Some	17.0%	17.6%	14.0%	16.7%	16.9%	17.4%	19.0%	18.6%	17.6%	17.6%
Many/All	12.0%	12.5%	14.9%	18.6%	12.5%	11.9%	13.2%	14.2%	13.8%	12.1%
Peer support	60.7%	62.9%	52.6%	48.7%	59.8%	70.1%	69.9%	60.0%	62.4%	69.2%
Family support	71.5%	70.8%	69.1%	56.3%	70.5%	66.1%	66.3%	49.9%	54.0%	64.6%
Teacher support	49.7%	50.1%	48.8%	46.1%	49.5%	45.3%	43.6%	43.6%	46.7%	45.2%
Classmates support	76.3%	76.0%	71.4%	70.2%	75.7%	65.2%	65.7%	55.7%	57.3%	64.3%

**Table 2:** Multivariable logistic regressions’ results, stratified by gender.

Country/Region of Birth	Current Smoking §	Current Alcohol Consumption §	Drunkenness §	Binge Drinking §	Cannabis §	Multiple Substance Use ^	At-Risk or Problem Gambling ^
BOYS							
Italy (native-born)	1	1	1	1	1	1	1
Western Countries	1.03 (0.69–1.54)	1.11 (0.76–1.61)	1.16 (0.79–1.71)	1.11 (0.78–1.57)	0.73 (0.44–1.21)	**1.44** (1.05–1.96)	0.86 (0.53–1.42)
Eastern European Countries	0.95 (0.53–1.70)	0.86 (0.52–1.42)	**1.58** (1.06–2.37)	1.15 (0.72–1.82)	1.12 (0.65–1.94)	1.14 (0.78–1.67)	**1.83** (1.04–3.22)
Non-Western/Non-European Countries	1.01 (0.69–1.49)	0.69 (0.45–1.06)	**0.50** (0.29–0.84)	0.75 (0.51–1.11)	1.03 (0.63–1.66)	0.95 (0.68–1.34)	**2.10** (1.29–3.42)
GIRLS							
Italy (native-born)	1	1	1	1	1	1	1
Western Countries	0.90 (0.62–1.30)	1.04 (0.67–1.60)	1.32 (0.83–2.09)	1.40 (0.89–2.18)	1.16 (0.68–1.97)	0.96 (0.66–1.40)	1.03 (0.38–2.83)
Eastern European Countries	1.15 (0.77–1.70)	**0.50** (0.29–0.85)	1.01 (0.61–1.66)	1.54 (1.00–2.37)	0.85 (0.50–1.45)	1.05 (0.75–1.49)	2.07 (0.93–4.64)
Non-Western/Non-European Countries	0.72 (0.50–1.03)	**0.55** (0.33–0.91)	1.19 (0.78–1.81)	1.26 (0.83–1.90)	1.14 (0.59–2.20)	**0.49** (0.35–0.70)	0.48 (0.17–1.30)

§ Adjusted for other substance use, peer substance use, and family, friends’, teachers’, and classmates’ support. ^ Adjusted for other substance use, and family, friends, teachers, and classmates’ support. Odds ratios and 95% confidence intervals are reported for each risk behavior considered (columns), entered one at a time as the model’s dependent variable, and for each area of origin (rows) setting Italy as the reference category. Significant odds ratios are presented in bold.

**Table 3:** Multivariable logistic regressions’ results, stratified by gender and migrant generation.

Country/Region of Birth	Generation	Current Smoking §	Current Alcohol Consumption §	Drunkenness §	Binge Drinking §	Cannabis §	Multiple Substance Use ^	At-Risk or Problem Gambling ^
BOYS								
Italy (native-born)		1	1	1	1	1	1	1
Western Countries	First	0.60 (0.17–2.13)	1.13 (0.32–4.05)	1.10 (0.29–4.13)	0.54 (0.15–2.00)	0.22 (0.03–1.58)	0.86 (0.24–3.02)	1.33 (0.35–5.02)
	Second	1.06 (0.69–1.62)	1.11 (0.75–1.63)	1.16 (0.78–1.73)	1.14 (0.80–1.63)	0.77 (0.46–1.27)	**1.47** (1.07–2.02)	0.84 (0.50–1.42)
Eastern European Countries	First	1.32 (0.48–3.68)	1.18 (0.53–2.66)	**2.08** (1.01–4.32)	1.00 (0.52–1.93)	1.81 (0.74–4.45)	1.30 (0.73–2.31)	1.52 (0.72–3.18)
	Second	0.79 (0.42–1.48)	0.71 (0.39–1.31)	1.35 (0.80–2.28)	1.27 (0.75–2.17)	0.82 (0.42–1.59)	1.04 (0.65–1.66)	2.04 (0.98–4.21)
Non-Western/Non-European Countries	First	0.46 (0.18–1.17)	1.08 (0.45–2.55)	0.58 (0.20–1.66)	0.74 (0.37–1.47)	1.22 (0.51–2.92)	0.99 (0.54–1.81)	**2.76** (1.02–7.43)
	Second	1.23 (0.78–1.93)	**0.59** (0.36–0.98)	**0.48** (0.27–0.85)	0.75 (0.47–1.22)	0.97 (0.56–1.65)	0.94 (0.63–1.40)	**1.92** (1.08–3.42)
GIRLS								
Italy (native-born)		1	1	1	1	1	1	1
Western Countries	First	0.74 (0.18–3.05)	0.36 (0.06–2.02)	1.54 (0.41–5.83)	0.69 (0.18–2.63)	3.60 (0.85–15.2)	0.58 (0.15–2.23)	NA *
	Second	0.90 (0.62–1.32)	1.07 (0.69–1.67)	1.31 (0.82–2.10)	1.43 (0.91–2.27)	1.09 (0.63–1.89)	0.99 (0.67–1.45)	1.10 (0.40–2.98)
Eastern European Countries	First	0.88 (0.51–1.52)	**0.33** (0.15–0.73)	0.93 (0.44–1.95)	**2.05** (1.04–4.02)	0.98 (0.49–1.99)	0.88 (0.52–1.48)	1.31 (0.38–4.54)
	Second	1.33 (0.79–2.24)	0.61 (0.32–1.16)	1.05 (0.58–1.91)	1.26 (0.74–2.13)	0.79 (0.39–1.60)	1.18 (0.75–1.86)	2.46 (0.95–6.39)
Non-Western/Non-European Countries	First	1.16 (0.57–2.37)	0.49 (0.16–1.55)	1.69 (0.68–4.18)	**2.71** (1.26–5.84)	1.18 (0.34–4.09)	0.62 (0.31–1.25)	**4.38** (1.26–15.3)
	Second	**0.64** (0.43–0.95)	**0.57** (0.33–0.97)	1.07 (0.66–1.75)	0.95 (0.60–1.49)	1.13 (0.57–2.24)	**0.46** (0.30–0.70)	**0.06** (0.02–0.20)

§ Adjusted for other substance use, peer substance use, and family, friends, teachers, and classmates’ support. ^ Adjusted for other substance use, and family, friends’, teachers’, and classmates’ support. * Analysis was not possible since no adolescents with this ethnic background presented the risk behavior. Odds ratios and 95% confidence intervals are reported for each risk behavior considered (columns), entered one at a time as the model’s dependent variable, and for each area of origin (rows) setting Italy as the reference category. Significant odds ratios are presented in bold.

## Data Availability

The data presented in this study are available in accordance with the Italian HBSC data access policy. Requests should be directed to paola.nardone@iss.it, a member of the National Centre for Disease Prevention and Health Promotion, Italian National Institute of Health.
